# How can we improve guideline use? A conceptual framework of implementability

**DOI:** 10.1186/1748-5908-6-26

**Published:** 2011-03-22

**Authors:** Anna R Gagliardi, Melissa C Brouwers, Valerie A Palda, Louise Lemieux-Charles, Jeremy M Grimshaw

**Affiliations:** 1 Departments of Surgery; and Department of Health Policy, Management and Evaluation; and Institute of Medical Science, Faculty of Medicine, University of Toronto, Toronto, Ontario, Canada; 2 Department of Oncology; Department of Clinical Epidemiology and Biostatistics, McMaster University, Hamilton, Ontario, Canada; 3 Department of Medicine; and Health Policy Management and Evaluation, University of Toronto, Guidelines Advisory Committee at the Centre for Effective Practice, Toronto, Ontario, Canada; 4 Department of Health Policy, Management and Evaluation; and Institute of Medical Science, Faculty of Medicine, University of Toronto, Toronto, Ontario, Canada; 5 Clinical Epidemiology; Department of Medicine, Centre for Best Practices, Institute of Population Health, University of Ottawa, Ottawa, Ontario, Canada

## Abstract

**Background:**

Guidelines continue to be underutilized, and a variety of strategies to improve their use have been suboptimal. Modifying guideline features represents an alternative, but untested way to promote their use. The purpose of this study was to identify and define features that facilitate guideline use, and examine whether and how they are included in current guidelines.

**Methods:**

A guideline implementability framework was developed by reviewing the implementation science literature. We then examined whether guidelines included these, or additional implementability elements. Data were extracted from publicly available high quality guidelines reflecting primary and institutional care, reviewed independently by two individuals, who through discussion resolved conflicts, then by the research team.

**Results:**

The final implementability framework included 22 elements organized in the domains of adaptability, usability, validity, applicability, communicability, accommodation, implementation, and evaluation. Data were extracted from 20 guidelines on the management of diabetes, hypertension, leg ulcer, and heart failure. Most contained a large volume of graded, narrative evidence, and tables featuring complementary clinical information. Few contained additional features that could improve guideline use. These included alternate versions for different users and purposes, summaries of evidence and recommendations, information to facilitate interaction with and involvement of patients, details of resource implications, and instructions on how to locally promote and monitor guideline use. There were no consistent trends by guideline topic.

**Conclusions:**

Numerous opportunities were identified by which guidelines could be modified to support various types of decision making by different users. New governance structures may be required to accommodate development of guidelines with these features. Further research is needed to validate the proposed framework of guideline implementability, develop methods for preparing this information, and evaluate how inclusion of this information influences guideline use.

## Background

Guidelines are syntheses of best available evidence that support decision making by clinicians, managers, and policy makers about the organization and delivery of healthcare, but continue to be underused. Numerous population-based studies demonstrate low compliance with guidelines produced by prominent government and professional agencies for chronic and acute conditions [1-7]. It has been proposed that for a condition such as cancer, a third of cases could be prevented, another third cured, and the remainder effectively treated if management consistently complied with existing guidelines [8]. Thus, it is imperative that we better implement guidelines.

Many existing implementation strategies have limited effectiveness and are not routinely applied outside of experimental research due to their cost and complexity [9-18]. As a result many guidelines are passively distributed [19-21]. Surveys of international guideline developers found that few developers implemented their own guidelines, had dedicated implementation staff, or evaluated use of their guidelines, and many believed that target users should be responsible for implementation [22-25]. Accountability for guideline implementation may differ by jurisdiction and organization depending on the structure of the healthcare system and how programs are funded. Hence, there is a need to develop broadly applicable strategies for implementing guidelines.

Observational studies have shown that use of guidelines is associated with the complexity of the recommended clinical action so a promising, but untested option is to alter guideline recommendations to make them more easily implementable by target users [26,27]. Implementability has been referred to as the characteristics of guideline recommendations that may enhance their implementation, and instruments have been developed to guide the formulation of implementable recommendations [28,29]. However, research suggests that including information within guidelines to assist users with implementation of the recommendations may promote greater understanding of how users are to accommodate the recommendations, which may stimulate confidence in capacity to practice the recommended behaviour, leading to greater intent to use guidelines and possibly actual use [30].

For example, in a systematic review of 256 studies, 41 of them found that lack of comprehensible structure and local applicability were barriers of guideline use [30]. Two randomized controlled trials (RCTs) examined the influence of guideline attributes on use. In one RCT, physicians of various specialties who received a guideline on electrodiagnostic tests (EDT) for patients with low back pain that was modified to include vignettes to illustrate use in patients with differing indications were more likely to use EDT appropriately compared with those who received the usual guideline [31]. Another RCT found that wording a guideline in behaviourally specific terms enhanced patient attitude about, confidence in ability to use, and intention to use the recommendations [32,33]. Furthermore, the information relevant to various guideline users may differ. Users include clinicians who deliver care, and managers and policy makers who must reconcile the competing interests of multiple stakeholders to make decisions about mobilizing organizational or system level resources [34]. Research suggests that individual clinicians value an easy-to-use format, evidence clarity and validity, details about competency and training requirements, and guidance on how to blend experience with evidence when applying the recommendations to individual patients, and engage patients in shared decision making [35-39]. Managers and policy makers want guidelines to summarize resource or policy implications, and be publicly available in different versions for various purposes [40,41].

It appears that guideline format and content may be important aspects of implementability that may influence use, and specific content may be required to support different types of decision making, including evidence-informed, experiential, shared, and resource allocation decision making. Including implementability information within guidelines to help users apply the recommendations represents a less-threatening, practice-relevant approach to guideline implementation compared with complex, costly, inconsistently effective implementation strategies often viewed negatively by guideline users [42]. It may be easier to modify the content and format of guidelines rather than the clinical complexity of the recommendations. Finally, this approach may be more feasible for guideline developers to integrate with the processes they already use to create guidelines regardless of health system or funding structure. Therefore, further investigation of how to make guidelines more implementable is warranted.

To date, there has not been a systematic analysis of guideline features that may improve their use. The purpose of this study was to create a taxonomy of these attributes, and assess whether current guidelines contain these features, thereby identifying ways in which guidelines could be modified to potentially improve their use. This implementability framework could inform the development of modified guidelines or adjunct products, and evaluation of how various attributes influence perceptions about, and use of guidelines, prior to more definitive testing of whether their inclusion indeed improves use.

## Methods

This study involved two key phases. The first phase was to develop an implementability framework of guideline format and content apart from clinical recommendations that are desired by users, or influence use of guidelines. The second phase was to use the framework assembled in phase one to examine the content of current practice guidelines, and refine or extend the framework.

### Development of an implementability framework

#### Approach

Given the lack of controlled and observational studies on this topic, the methods were based on a modified meta-narrative approach [43]. The meta-narrative approach is more suitable than a systematic review for conceptually examining literature that may be limited in quantity and quality, and vary in disciplinary focus and study design. It involves periodic input from a multidisciplinary research team to define the objectives and interpret the findings from a variety of conceptual perspectives. In this case, we used a modified approach that focused on healthcare literature rather than other disciplines, but were inclusive of a variety of study designs.

#### Data collection

One individual with health librarian training and experience conducting different types of reviews (ARG) performed searches in MEDLINE and EMBASE from 1996 to August 2009. We purposely used a broad search strategy based on few terms (practice guidelines as topic AND guideline adherence AND attitude of health personnel or decision making, organizational or policy making) knowing that sensitivity and specificity would be limited. Two individuals independently selected eligible items. Eligible titles included empirical quantitative (meta-analyses, surveys, observational studies, randomized trials) or qualitative (reviews/conceptual analyses, interviews, focus groups) studies published in English language peer-reviewed journals describing guideline features desired by, or influencing the knowledge, decision making, or behaviour of health professionals. Studies were ineligible if they focused on guideline-informed tools such as clinical pathways; guidelines for non-medical interventions; clinical effectiveness of medical interventions; involved students, trainees, or patients as participants; investigated guideline use without examining views about guideline features that influenced use; evaluated interventions to promote guideline use; concluded that guideline features could be improved to promote their use without evaluating those features; or were in the form of abstracts, letters, commentaries, or editorials. All items selected by at least one individual were retrieved, and one individual extracted data. Quality assessment of studies was not undertaken to be inclusive of all relevant implementability elements.

#### Data analysis

Desirable or influential features potentially associated with guideline use were annotated in eligible studies, then tabulated. This tabulated list included the features of guidelines identified in each study as desirable or influencing guideline use. From this list, common items were categorized and defined. Findings were reviewed independently, and as a group by the study team, which included individuals with clinical, management, and research perspectives; grounding in the disciplines of knowledge translation/implementation science, psychology, and organizational behaviour; and experience in guideline development, guideline implementation, and performance improvement. The research team met in person and by teleconference to review and refine the draft framework. This largely involved minor edits to domain definitions. The draft framework was used to guide content analysis of guidelines, which expanded the number of elements in framework domains. This extended framework was reviewed and refined by the research team in person and by teleconference.

### Application of the implementability framework

#### Approach

Manifest content analysis was used to examine guidelines for the presence of implementability elements. This is a method that describes explicit content as reported in written, verbal, or visual communication qualitatively and/or quantitatively, without interpretation of its underlying meaning [44]. We selected a directed approach, which seeks to validate and extend elements in a framework [45]. This means data are coded using elements in the draft framework, and data that cannot be coded are analyzed to assess if they represent a new element.

#### Sampling

Individual guidelines were chosen as the unit of analysis. Guidelines on topics representing a high burden of illness in primary (diabetes, hypertension) and institutional care (chronic ulcer, heart failure) were selected from among those evaluated by the Guidelines Advisory Committee (GAC, http://www.gacguidelines.ca), a program that systematically appraised and summarized guidelines. Eligible guidelines included all those identified by GAC using a comprehensive search strategy and judged by trained experts using the Appraisal of Guidelines Research and Evaluation (AGREE) instrument to be high quality that covered comprehensive management of these conditions and were publicly available [46].

#### Data collection

Full versions of selected guidelines and adjunct products were retrieved from sponsor web sites. A form was developed to extract content from each guideline according to the implementability framework. Round one extraction was performed by ARG. This produced an expanded, revised framework, used by ARG to again extract data from each guideline. A research assistant independently reviewed the features in all guidelines, and a physician (VAP) independently reviewed coding of the elements for two guidelines on each clinical topic. ARG met with both independent reviewers to compare findings and resolve differences through discussion.

#### Data analysis and interpretation

Extracted data was tabulated. The presence of implementability elements within sampled guidelines was described using summary statistics including number, proportion, and mean or median. Detailed content was analyzed using Mays' narrative review method, based on verbatim reporting, rather than statistical summary or conceptual analysis of information [47]. Data were examined to discuss the number of guidelines addressing each element overall and by topic, thereby identifying opportunities for modifying guideline format or content to enhance implementability. Findings were reviewed by the research team in person and by teleconference.

## Results

A total of 18 studies were reviewed from among 1,348 (441 MEDLINE, 907 EMBASE) identified by the literature search (Table 1). The vast majority of literature search results were ineligible because they evaluated the clinical effectiveness of medical interventions or interventions to promote guideline use. Eligible studies included one RCT, one observational study, four systematic reviews, three surveys, two modified Delphi studies, and six qualitative studies involving either focus groups or interviews. Based on features desired by, or influencing guideline use among primarily physicians, a preliminary taxonomy of eight implementability domains emerged, including adaptability, usability, validity, applicability, communicability, accommodation, implementation, and evaluation (Table 2).

**Table 1:** Studies describing guideline features that may influence use

Study	Design	Guideline features encouraging use
Brouwers 2009 Canada [48]	Survey of 756 physicians of various specialties between 1999 and 2005 on intended use of 84 cancer guidelines yielding 4,091 surveys	Strong supporting evidence, flexibility of recommendations to local context

Wakkee 2008 Netherlands [49]	Questionnaire of 261 dermatologists on characteristics of specific guideline	Concise recommendations

Nuckols 2008 United States [39]	Modified Delphi panel of 11 physicians of various specialties	Strong supporting evidence, flexibility of recommendations to patient needs and preferences

Francke 2008 Netherlands [7]	Meta-review of 12 systematic reviews on guideline implementation:	
	1. 41 cross sectional pre-/post-test studies or controlled trials	Easily accessible, strong supporting evidence, explicit resource implications, flexibility of recommendations to local
	2. 76 survey and qualitative studies	
	3. 91 randomized, cross-over, balanced incomplete block design, controlled before/after, interrupted times series studies	context, concise recommendations
	4. 61 mixed methods studies with focus on randomized or controlled trials	
	5. 23 studies of various quantitative designs	
	6. 235 randomized or controlled trials, controlled before/after or interrupted time series designs	
	7. 40 randomized or controlled trials or before/after studies	
	8. 15 randomized or controlled trials, pre-/post-test studies and one systematic review	
	9. 59 studies of various quantitative or qualitative or mixed design	
	10. 6 randomized controlled trials, time series or before/after studies and 8 studies of mixed design	
	11. 18 ranodmized or controlled trials, before/after or interrupted time series studies	
	12. 20 randomized or controlled trials, case series or case reports	

Cochrane 2007 United States [30]	Systematic review of 256 studies of guideline implementation (178 surveys, 16 focus group studies, 18 interview studies, 44 mixed methods studies)	Easily accessible, strong supporting evidence, flexibility of recommendations to local context, concise recommendations

Carlsen 2007 Norway [50]	Qualitative analysis of six focus groups involving 27 general practitioners	Trustworthy, suit patients, recommended action is feasible

Carlsen 2007 Norway [42]	Systematic review of 12 qualitative studies (7 focus group studies, 5 interview studies) evaluating general practitioner attitudes about guidelines	Authorship familiarity, flexibility of recommendations to patient needs and preferences, short and concise, include patient leaflets

Jones 2007 Canada [51]	Qualitative analysis 28 interviews with physicians and nurses in four intensive care units	Easily accessible, accompanying tools such as checklists, strong supporting evidence, concise recommendations

Thomason 2007 United States [52]	Survey and focus groups with 60 physicians and nurses who attended a national conferences	Strong supporting evidence, concise recommendations

Sinuff 2007 Canada [53]	Qualitative analysis of interviews with 30 physicians and nurses at one hospital	Easily accessible, accompanying tools such as algorithms or pocket cards, concise recommendations

McKinlay 2004 New Zealand [54]	Qualitative analysis of interviews with 13 general practitioners from five sites	Authorship familiarity, variety of print and electronic formats

Shiffman 2003 United States [55]	Modified Delphi process involving representatives from 22 organizations active in guideline development	Explicit resource implications, suggestions for auxiliary documents for providers or patients, evaluative data collection tools

Price 2001 United States [56]	Discourse analysis of laboratory study using clinical scenarios and guidelines of different formats involving three general practitioners and three endocrinologists	Algorithmic guidelines were useful for clinical problem solving, textual guidelines were useful for learning

Vinker 2000 Israel [57]	Questionnaire of 293 general practitioners and family physicians participating in educational programs over two months	Strong supporting evidence, flexibility of recommendations to patient needs and preferences, concise recommendations

Harris 2000 United States [42]	Questionnaire and focus groups with an undisclosed sample drawn from 304 general practitioners based at 16 sites	Accompanying tools such as checklists and standard orders, summaries such as algorithms or diagrams, navigational support such as color-coded tabs, evaluative data collection tools, accessible by computer, information guides for patients

Shekelle 2000 United States [31]	Randomized controlled trial of questionnaire on intent to use guidelines among 545 general internists, neurologists and physical medicine specialists who received usual guideline or guideline modified with clinical vignettes	Clinical vignettes describing application of guidelines according to patient needs and preferences

Cabana 1999 United States [58]	Systematic review of 76 journal articles on barriers to guideline adherence among physicians	Strong supporting evidence, authorship familiarity, easily accessible, concise recommendations, flexibility of recommendations to patient needs and preferences

Grol 1998 Netherlands [37]	Observational study involving 12,880 decisions made by 61 general practitioners based on 12 guidelines with various attributes rated by participants	Strong supporting evidence, concise recommendations, explicit resource implications

**Table 2:** Initial framework of guideline implementability

Domain	Definition
Adaptability	The guideline is available in a variety of versions for different users or purposes.

Usability	Content is presented, organized, or formatted to enhance the ease with which the guideline can be employed.

Validity	Evidence is summarized and presented such that its quantity and quality are apparent, and it can be easily reviewed, understood, and interpreted.

Applicability	Contextual or supplementary clinical information is provided by which to interpret and apply the recommendations for individual patients.

Communicability	Information is included to support discussions with patients, or patient involvement in decision making.

Accommodation	Costs, resources, competencies and training, technical specifications, and anticipated impact required to accommodate use are identified.

Implementation	Strategies for identifying barriers of use, and selecting, planning, and applying promotional strategies are described.

Evaluation	Performance measures for audit or monitoring are included.

Based on the implementability framework, data were extracted from 20 guidelines on the management of diabetes (n = 8), hypertension (n = 4), leg ulcer (n = 3) and heart failure (n = 5) from eight different countries, including Australia (n = 2), Brussels (n = 1), Canada (n = 4), the Netherlands (n = 1), New Zealand (n = 1), Singapore (n = 1), the United Kingdom (n = 4), and United States (n = 6) (Table 3). Most were produced by professional associations or government agencies (75%). The final framework derived through content analysis of guidelines included 22 elements organized within eight domains (Table 4).

**Table 3:** Guidelines reviewed by type of organization and clinical topic

Type of organization	Overall	Diabetes	Hypertension	Leg Ulcer	Heart Failure
Academic	1	1	---	---	---

Government	6	2	2	1	1

Expert panel or consortium	2	1	---	1	---

Professional association	9	3	2	1	3

Private, nonprofit	2	1	---	---	1

Total	20	8	4	3	5

**Table 4:** Final framework of guideline implementability

Domain	Element	Examples
Usability	Navigation	Table of contents
	
	Evidence format	Narrative, tabulated or both
	
	Recommendation format	Narrative, graphic (algorithms) or both; Recommendation summary (single list in full or summary version)

Adaptability	Alternate versions	Summary (print, electronic for PDA); Patient (tailored for patients/caregivers); Published (journal)

Validity	Number of references	Total number of distinct references to evidence upon which recommendations are based
	
	Evidence graded	A system is used to categorize quality of evidence supporting each recommendation
	
	Number of recommendations	Total number of distinct recommendations (sub-recommendations considered same)

Applicability	Individualization	Clinical information (indications, criteria, risk factors, drug dosing) that facilitates application of the recommendations explicitly highlighted as tips or practical issues using sub-titles or text boxes, or summarized in tables and referred to in recommendations or narrative contextualizing recommendations

Communicability	Patient education or involvement	Informational or educational resources for patients/caregivers, questions for clinicians to facilitate discussion, or contact information (phone, fax, email or URL) to acquire informational or educational resources

Accommodation	Objective	Explicitly stated purpose of guideline (clinical decision making, education, policy, quality improvement)
	
	Users	Who would deliver/enable delivery of recommendations (individuals, teams, departments, institutions, managers, policy makers, internal/external agents), who would receive the services (patients/caregivers)
	
	User needs/values	Identification of stakeholder needs, perspectives, interests or values
	
	Technical	Equipment or technology needed, or the way services should be organized to deliver recommendations
	
	Regulatory	Industrial standards for equipment or technology, or policy regarding their use
	
	Human resources	Type and number of health professionals needed to deliver recommended services
	
	Professional	Education, training or competencies needed by clinicians/staff to deliver recommendations
	
	Impact	Anticipated changes in workflow or processes during/after adoption of recommendations
	
	Costs	Direct or productivity costs incurred as a result of acquiring resources or training needed to accommodate recommendations, or as a result of service reductions during transition from old to new processes

Implementation	Barriers/facilitators	Individual, organizational, or system barriers that are associated with adoption
	
	Tools	Instructions, tools or templates to tailor guideline/recommendations for local context; Point-of-care templates/forms (clinical assessment, standard orders)
	
	Strategies	Possible mechanisms by which to implement guideline/recommendations

Evaluation	Monitoring	Suggestions for evaluating compliance with organization, delivery and outcomes of recommendations, including program evaluation, audit tools, and performance measures/quality indicators

Format elements that may facilitate guideline use are summarized in Table 5. One-half of the guidelines were published in journals or available in a summary version, and one-quarter were available as downloadable digital or patient versions. Many were very large documents with median pages of 72.5 (range 21.0 to 878.0), median number of references of 230.5 (range 15.0 to 3,487.0), and median number of recommendations of 41.5 (range 8.0 to 214.0). Most featured a table of contents (75.0%), and just over one-half included a recommendation summary (55.0%) or algorithm (65.0%). Nearly all guidelines used an evidence grading system (95.0%). Few summarized the evidence in tabular format (25.0%).

**Table 5:** Format elements of reviewed guidelines

Domain/Element	Statistic	Overall (n = 20)	Diabetes (n = 8)	Hypertension (n = 4)	Leg Ulcer (n = 3)	Heart Failure (n = 5)
Adaptability						

Journal version	n (%)	10 (50.0)	4 (50.0)	3 (75.0)	0 (0.0)	3 (60.0)

PDA version	n (%)	5 (25.0)	3 (37.5)	1 (25.0)	0 (0.0)	1 (20.0)

Short version	n (%)	9 (45.0)	0 (0.0)	3 (75.0)	2 (66.7)	4 (80.0)

Patient version	n (%)	4 (20.0)	0 (0.0)	2 (50.0)	0 (0.0)	2 (40.0)

Usability						

Table of contents	n (%)	15 (75.0)	6 (75.0)	2 (50.0)	3 (100.0)	4 (80.0)

Number of pages	mean	120.2	199.5	45.8	60.7	88.4
	
	med	72.5	95.5	46.0	46.0	80.0
	
	min	21.0	21.0	39.0	21.0	25.0
	
	max	878.0	878.0	52.0	115.0	163.0

Number of recommendations	mean	71.7	120.8	10.5	41.3	60.4
	
	med	41.5	126.5	9.5	39.0	43.0
	
	min	8.0	24.0	8.0	20.0	9.0
	
	max	214.0	214.0	15.0	65.0	118.0

Recommendation summary	n (%)	11 (55.0)	3 (37.5)	4 (100.0)	2 (66.7)	2 (40.0)

Recommendation algorithm	n (%)	13 (65.0)	2 (25.0)	4 (100.0)	2 (66.7)	5 (100.0)

Validity						

Number of references	mean	452.0	849.9	128.8	111.7	278.2
	
	med	230.5	247.0	80.0	83.0	252.0
	
	min	15.0	15.0	24.0	72.0	218.0
	
	max	3,487.0	3,487.0	331.0	180.0	347.0

Evidence graded	n (%)	19 (95.0)	7 (85.5)	4 (100.0)	3 (100.0)	5 (100.0)

Evidence format	narrative	15 (75.0)	6 (75.0)	4 (100.0)	2 (66.7)	3 (60.0)
	
	narrative + tabular	5 (25.0)	2 (25.0)	---	1 (33.3)	2 (40.0)

Content elements that may facilitate guideline use are summarized in Table 6. Clinical considerations by which to individualize recommendations were available in most guidelines (90.0%). For many guidelines this largely consisted of tables that summarized diagnostic or risk criteria, pharmacologic dosing, indications for treatment or referral, and management options (75.0%). All four hypertension guidelines included specific skill-based instructions for measuring blood pressure. Some guidelines featured sections explicitly labelled considerations for either special populations (two diabetes guidelines) or by health system capacity (one diabetes guideline). Two heart failure guidelines graphically highlighted considerations within text boxes or balloons labelled practice points or tips. Less than one-half of the guidelines included information to educate or engage patients (50.0%). Of these, five provided information to help clinicians discuss relevant issues with patients, two included information sheets for patients, and seven provided contact information (phone number or web site) where information for patients could be obtained.

**Table 6:** Content elements of reviewed guidelines

Domain/Element	Overall (n = 20)		Diabetes (n = 8)		Hypertension (n = 4)		Leg Ulcer (n = 3)		Heart Failure (n = 5)	
	
	n	%	n	%	n	%	n	%	n	%
Applicability										

Individualization	18	90.0	6	75.0	4	100.0	3	100.0	5	100.0

Communicability										

Patient informed care	10	50.0	4	50.0	2	50.0	1	33.3	3	60.0

Accommodation										

Objectives:										
Clinical	20	100.	8	100.	4	100.0	3	100.0	5	100.0
Education	1	0	1	0	---	---	---	---	---	---
Policy	---	5.0	---	12.5	---	---	---	---	---	---
Quality improvement	2	--- 10.0	1	--- 12.5	---	---	---	---	---	---
Users	12	60.0	5	62.5	1	25.0	2	66.7	4	80.0

User needs/values	0	0.0	0	0.0	0	0.0	0	0.00	0	0.0

Technical	9	45.0	3	37.5	1	25.0	1	33.3	4	80.0

Regulatory	3	15.0	0	0.0	3	75.0	0	0.0	0	0.0

Human resources	1	5.0	1	12.5	0	0.0	0	0.0	0	0.0

Professional	4	20.0	0	0.0	1	25.0	2	66.7	1	20.0

Impact	0	0.0	0	0.0	0	0.0	0	0.0	0	0.0

Costs	0	0.0	0	0.0	0	0.0	0	0.0	0	0.0

Implementation										

Barriers	3	15.0	1	12.5	2	20.0	0	0.0	0	0.0

Tailoring instructions	2	10.0	0	0.0	0	0.0	2	66.7	0	0.0

Point-of-care tools/forms	6	30.0	3	37.5	0	0.0	2	66.7	1	20.0

Implementation strategies	9	45.0	4	50.0	1	25.0	2	66.7	2	40.0

Evaluation										

Evaluation instructions	0	0.0	0	0.0	0	0.0	0	0.0	0	0.0

Performance measures	10	50.0	4	50.0	2	50.0	2	66.7	2	40.0

No guidelines identified stakeholder needs or values, or costs or impact associated with use. Few included technical (45.0%), regulatory (15.0%), human resources (5.0%), or professional competency (20.0%) information required to accommodate guideline use. When included, this content was generally limited in detail. For example, technical guidance included: 'organization of care to deliver the above recommendations is largely concerned with putting registration, recall and record systems in place to ensure care delivery occurs for all people with diabetes, and having the healthcare professionals trained and available (D12)' or 'multidisciplinary care programs improve patients' quality of life, satisfaction with care, and the risk of unplanned hospitalization for heart failure (HF23).' Regulatory instructions included: 'blood pressure instruments must be properly validated and regularly recalibrated according to manufacturer instructions (H04).' Guidance for human resources included: 'interdisciplinary team comprised of family physician, diabetes educators (nurse, dietician), and community health support (D15).' Professional competency criteria included: 'compression bandages should be applied by a practitioner who has received training in their application (LU05).'

One-half of the guidelines included performance measures that could be used to monitor recommended clinical care. While 45.0% mentioned the need to actively promote guideline use, none thoroughly described how to undertake or evaluate this process. For example: 'implementation may be supported by a variety of activities including continuing education and training, and clinical audit (LU07)' or 'implementation programs are needed because it has been shown that the outcome of a disease may be favourably influenced by thorough application of clinical recommendations (HF26).' Less than a third included templates such as order forms or assessment checklists (30.0%), and fewer than this offered cursory instructions for identifying barriers of use (15.0%) or tailoring the guidelines to suit local circumstances (2.0%).

## Discussion

Relatively few studies published over the last 15 years specifically examined guideline features desired by, or associated with use among health professionals, most of these focused on physicians, and it does not appear that studies were informed by preceding research to build a cumulative body of knowledge. Considerable research has examined other factors influencing guideline use such as physician and organizational characteristics, but these studies were not eligible for this review, nor were numerous studies that examined general attitudes to guidelines on specific clinical topics. Review of 18 eligible studies revealed several features related to format or content that may positively influence guideline use, and this was expanded by reviewing the content of high quality international guidelines on various clinical topics. Most guidelines we examined contained a large volume of graded evidence and numerous tables featuring complementary clinical information to the point of being cumbersome, despite the presence of navigational features such as tables of contents. Few contained additional features specified by users or suggested by research to improve guideline use. Guideline use could potentially be improved by developing alternate versions for different purposes, incorporating summaries of evidence and recommendations, including information to facilitate interaction with and involvement of patients, outlining resource implications, and describing how to locally plan, promote, and monitor guideline use. There were no consistent trends by guideline topic.

Our findings simply suggest that more guidelines could be modified to include implementability content, but it remains unclear how various implementability features might influence guideline use. A recent analysis recommended that the reliability, relevance, and readability of knowledge resources be improved to support evidence-based decision making [48]. Evidence is just one of several factors that inform decisions about guideline use [49]. In reality, clinicians must often draw upon expertise and experience to consider what is best for and desired by those receiving care, but have expressed uncertainty about how to balance evidence with professional judgment and patient preferences, and the need for guidance to support these decisions [50,51]. Furthermore, clinical decisions about guideline use are influenced by the availability and mobilization of organizational or system level resources, which are governed by managers and policy makers who must reconcile the competing interests of multiple stakeholders [34]. Further insight could be gained by drawing upon decision science to examine the cognitive processes underlying guideline use. Considerable research has established that humans are not rational decision makers who identify alternative options, compare them on the same set of evaluative dimensions, and generate probability and utility estimates for different courses of action [52]. Instead, it appears that a combination of intuitive (based on experience) and analytic (based on mental simulation) mechanisms are employed [53]. This is particularly true in 'naturalistic' situations where decisions are complex; the quantity of information may be large or its implications ambiguous; goals may be shifting, poorly defined, or competing; and decisions have high stakes and are made within a dynamic environment under time constraints, as is true of the healthcare sector [54]. It has been suggested that guidelines include content that mediates decisions among different stakeholders in a manner consistent with these cognitive processes [55]. Thus, elements in the proposed framework may have impact on two dimensions: support for different types of decision making (evidence-informed, experiential, shared, allocation/policy) by providing particular information and/or tools, and support for different types of decision-making processes (intuitive, analytic) by making explicit the options for, and implications of alternate choices. This may influence attitudes about guideline relevance and confidence in choosing a course of action, which may be associated with use [56]. While the concept of implementability is not new, the proposed framework is unique because it includes features that may be relevant not only to individuals, but to the managers and policy makers that govern the environment within which individuals function, and because it offers a novel way to improve guideline use by considering how to support different types and processes of decision making [28,57,58].

Interpretation of the findings may be limited in several ways. We studied guidelines relevant to primary and institutional care. Other guidelines relevant to specialty care may differ in their implementability characteristics. However, while we reviewed few guidelines, they were specifically selected to represent different topics, countries, and types of developer. Each element may not have been relevant to all guidelines reviewed, but this exercise serves as an exploratory, baseline effort to develop the framework according to content available in a range of guidelines. The literature on this topic is sparse, and referred to conceptually in a variety of ways and therefore not consistently indexed in literature databases; the search strategy used was purposely broad in an attempt to identify all relevant studies, but it may not have retrieved all studies describing guideline features desired by, or influencing the behavior of health professionals. We are currently in the process of conducting a systematic conceptual review of theoretical and empirical research on the mechanisms by which implementability elements influencing decision making about guideline use. Still, by assembling a rudimentary implementability framework that was expanded by review of guideline content, numerous opportunities were revealed for potentially improving guideline development and use.

Prior to testing these hypotheses, practical issues must be considered. Robust methods by which to operationalize concepts more specifically to enable accurate data capture would require further development. New governance structures may be required to accommodate the development of guidelines with these features. Future research should validate the framework by applying it to different types of guidelines, and by soliciting feedback from guideline development and implementation experts, clinicians, managers, policy makers, and patients/caregivers to further clarify and expand on its elements. Research is also required to examine precisely how the elements of implementability influence guideline use. Based on an expanded stakeholder-defined implementability framework, the cost-effectiveness of tailored guidelines or adjunct products could be established by examining short-term outcomes predictive of guideline use such as recall, attitude to, confidence in, and adoption intention, then long-term objective outcomes reflecting the adoption of processes and associated patient care outcomes.

## Conclusions

Numerous opportunities were identified by which guidelines could be modified to potentially facilitate their use. New governance structures may be required to accommodate development of guidelines with these features. Further research is needed to validate the proposed framework of guideline implementability, develop methods for preparing this information, and evaluate how inclusion of this information influences guideline use.

## Competing interests

The authors declare that they have no competing interests.

## Authors' contributions

ARG conceptualized and designed this study and obtained funding. She performed primary data collection, analysis, interpretation and report writing. MCB, LLC and JMG assisted with design of this study and data interpretation. VAP assisted with design of this study, independently reviewed data extracted from guidelines, and assisted with interpretation. All co-investigators contributed to report writing, and read and approved the final version of this manuscript.
